# Role of Cationic Side Chains in the Antimicrobial Activity of C18G

**DOI:** 10.3390/molecules23020329

**Published:** 2018-02-04

**Authors:** Eric M. Kohn, David J. Shirley, Lubov Arotsky, Angela M. Picciano, Zachary Ridgway, Michael W. Urban, Benjamin R. Carone, Gregory A. Caputo

**Affiliations:** 1 Department of Chemistry and Biochemistry, Rowan University, Glassboro, NJ 08028, USA; kohne2@students.rowan.edu (E.M.K.); shirleyd9@students.rowan.edu (D.J.S); larotsky@upenn.edu (L.A.); ap775@cornell.edu (A.M.P.); zachary.ridgway@stonybrook.edu (Z.R.); urbanm@rowan.edu (M.W.U.); 2 Thomas N. Bantivoglio Honors Concentration, Rowan University, Glassboro, NJ 08028, USA; carone@rowan.edu; 3 Department of Molecular and Cellular Biosciences, Rowan University, Glassboro, NJ 08028, USA; carone@rowan.edu

**Keywords:** antimicrobial peptides, fluorescence, lipid binding, non-natural amino acids

## Abstract

Antimicrobial peptides (AMPs) have been an area of great interest, due to the high selectivity of these molecules toward bacterial targets over host cells and the limited development of bacterial resistance to these molecules throughout evolution. The peptide C18G has been shown to be a selective, broad spectrum AMP with a net +8 cationic charge from seven lysine residues in the sequence. In this work, the cationic Lys residues were replaced with other natural or non-proteinogenic cationic amino acids: arginine, histidine, ornithine, or diaminopropionic acid. These changes vary in the structure of the amino acid side chain, the identity of the cationic moiety, and the pK_a_ of the cationic group. Using a combination of spectroscopic and microbiological methods, the influence of these cationic groups on membrane binding, secondary structure, and antibacterial activity was investigated. The replacement of Lys with most other cationic residues had, at most, 2-fold effects on minimal inhibitory concentration against a variety of Gram-positive and Gram-negative bacteria. However, the peptide containing His as the cationic group showed dramatically reduced activity. All peptide variants retained the ability to bind lipid vesicles and showed clear preference for binding vesicles that contained anionic lipids. Similarly, all peptides adopted a helical conformation when bound to lipids or membrane mimetics, although the peptide containing diaminopropionic acid exhibited a decreased helicity. The peptides exhibited a wider variety of activity in the permeabilization of bacterial membranes, with peptides containing Lys, Arg, or Orn being the most broadly active. In all, the antibacterial activity of the C18G peptide is generally tolerant to changes in the structure and identity of the cationic amino acids, yielding new possibilities for design and development of AMPs that may be less susceptible to immune and bacterial recognition or in vivo degradation.

## 1. Introduction

The continued development and spread of antibiotic resistance is a major concern, with the World Health Organization (WHO) and the United States Centers for Disease Control (CDC) identifying antibiotic resistance as a major health threat in the coming years [1,2]. Patients who become infected with resistant strains of bacteria often require longer hospital stays or treatments, therefore increasing the total cost of therapy [3,4]. Due to the rapid life cycle of bacteria and pursuant random mutations or genetic alterations, as well as improper use of existing antibiotics, bacteria have many advantages in acquiring resistant phenotypes. Additionally, many pathogenic bacteria can form biofilm structures that create an additional permeability barrier to antimicrobial therapeutics [5,6].

The rise of antimicrobial resistance not only underscores the need for development of new antimicrobial compounds, but also highlights the necessity to develop new compounds with lower potentials for development of resistance. Many approaches have been taken in the development of new or improved antimicrobials, including combinatorial therapies [7,8], biomolecule mimetics [9,10], natural products [11,12], synthetic polymers [13,14], metals and metal complexes [15], quorum sensing inhibitors [16,17], and antimicrobial peptides (AMPs) [18]. AMPs have been an area of extensive study, as they are relatively straightforward to synthesize and characterize, biologically and evolutionarily diverse, and show little evidence of resistance development throughout evolution. Generally, AMPs are cationic, amphiphilic peptides that exhibit high selectivity for bacterial targets over host cells. This selectivity arises from the net positive charge on the AMPs and the net negative surface charge of the bacterial target cells, as opposed to the net neutral charge of the mammalian or host cell membrane. However, there is little in the way of consensus sequences or structural motifs that are conserved throughout this class of peptides [19]. Nonetheless, cationic, helical AMPs are among the most widely found in nature, and widely studied [20,21]. These molecules are relatively short (less than 40aa), contain a mix of hydrophobic and cationic amino acids, and often adopt a facially-amphiphilic helical structure when bound to the lipid bilayer or bacterial membrane. The consensus in the literature is that these AMPs act by permeabilizing the bacterial membrane, but there are reports of other mechanisms as well, including DNA damage [22] and oxidative stress or damage [23]. There are also numerous reports of AMPs acting through an immunomodulatory role [24,25]. The proposed mechanism of membrane disruption is through the partitioning of the hydrophobic helical face into the bilayer core, disrupting lipid packing and creating transient pores that allow leakage of content across the membrane [26].

One interesting example of the helical AMPs is the peptide C18G. This peptide was originally derived from the C-terminal 13 amino acids of platelet factor IV and modified to enhance the activity [27,28]. The C18G peptide exhibits broad spectrum activity, with high selectivity for bacterial cells over host cells, and generally low toxicity to host cells. The peptide sequence is inherently interesting, as the majority of the 18 residues are either Leu or Lys, with all of the cationic charge arising from Lys and all of the hydrophobic character coming from Leu. Subsequently, C18G was found to interact with several two-component signaling systems in bacteria, identifying these proteins as potential AMP sensors in addition to the original ligands Mg^+2^ [29,30], ferric iron (Fe^+3^), aluminum (Al^+3^), or pH [31]. More recently, the role of the hydrophobic amino acids in C18G was investigated by replacing all the Leu residues with other hydrophobic amino acids. This study demonstrated that hydrophobicity alone does not determine the activity of the peptides, and that the structure of the hydrophobic amino acid side chain plays a significant role in membrane permeabilization activity [32]. 

Despite the previous work on C18G and AMPs in general, there is still no consensus on the molecular determinants of activity. While the hydrophobic groups influence the activity of the peptide, there have been no reports in the literature investigating the role of the cationic amino acids in the peptide behavior. In the work presented here, the Lys residues were systematically replaced with either arginine, histidine, ornithine (Orn, O), or diaminopropionic acid (Dap, X). The Arg and His residues were chosen as they are the other naturally occurring cationic amino acids, while Orn and Dap were selected as they retain the primary amino group of Lys, but alter the side chain length, potentially altering the membrane penetration depth or the 3-D topography of the peptide when bound to the membrane surface. Using a combination of fluorescence spectroscopy and circular dichroism spectroscopy, the interactions of the peptides with model lipid vesicles were investigated. The membrane permeabilizing activity of the peptides was then investigated using enzyme based bacterial membrane permeabilization assays, flow cytometry, and red blood cell hemolysis experiments. 

## 2. Results

### 2.1. Peptide Sequences and Structure

**Figure 1:**
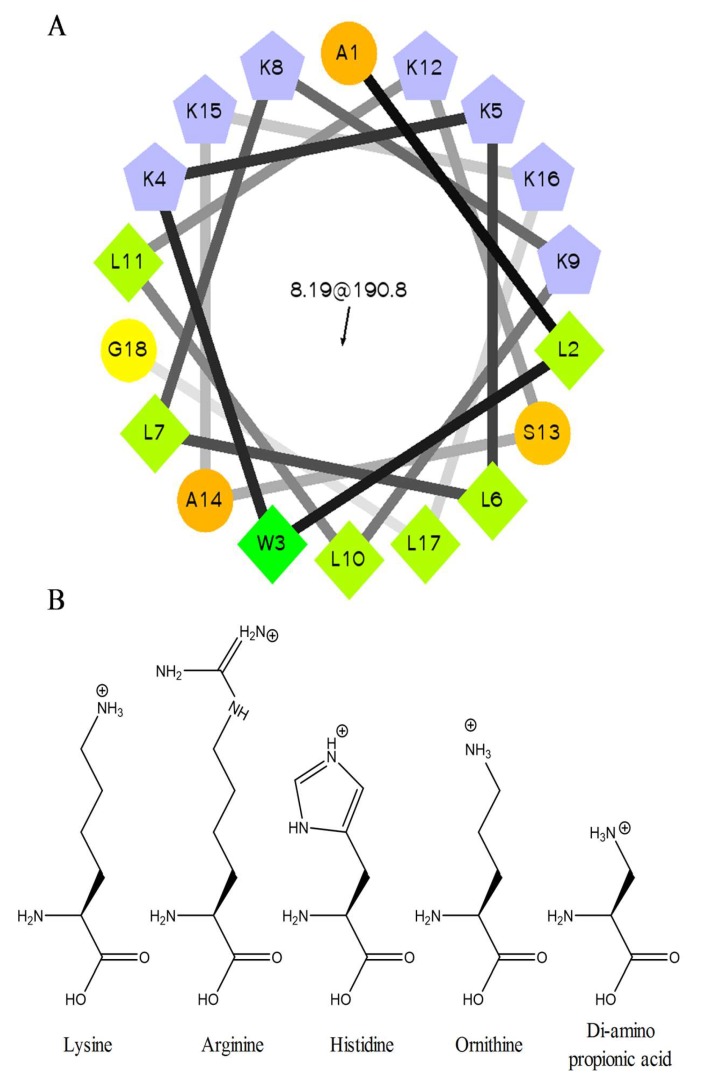
Peptide and amino acid structures. (**A**) The C18G sequence is depicted in a helical wheel diagram where cationic amino acids are blue, hydrophobes are green, and polar-uncharged are orange. (**B**) The chemical structures of the cationic amino acids used in this study are shown at the bottom of the figure. The helical wheel diagram was created using software created by Don Armstrong and Raphael Zidovetzki. Version: Id: wheel.pl,v 1.4 2009-10-20 21:23:36 don Exp.

The original C18G sequence and a number of sequence variants have been previously shown to adopt an ⍺-helix when bound to bilayers or membrane mimetics [32]. A helical wheel diagram of C18G is shown in Figure 1A. The helical conformation of C18G, and all of the cationic variants in this report, adopt a facially amphiphilic helical structure, with cationic residues segregated to one face of the helix and the hydrophobic groups segregated to the opposite face of the helix. As the focus of this work is on the cationic amino acids, the structures of the cationic side chains are shown in Figure 1B, and peptide sequences in this study are shown in Table 1. 

### 2.2. Antibacterial Activity Assays

The cationic variants of C18G were first assessed for the ability to inhibit bacterial growth in the standard minimum inhibitory concentration (MIC) assay. The results of the MIC assay are shown in Table 2. There were several notable results from this analysis, the foremost being that the C18G-Dap peptide exhibited at most a 4-fold increase in MIC (against *Staphylococcus aureus* (*S. aureus)*) but in two cases, identical MICs (*Bacillus subtilis* (*B. subtilis)* and *Pseudomonas aeruginosa* (*P. aeruginosa)*) to the parent sequence. Interestingly, the C18G-His peptide showed significantly reduced antibacterial activity against most of the strains tested, but very similar MIC values to the other peptides when challenged with *Escherichia coli* (*E. coli)* or *B. subtilis*. The minimal bactericidal concentration (MBC) values, when able to be determined, were no more than 2-fold higher than the MIC value in all cases, except C18G-His against *B. subtilis*. These results indicate that the peptides are acting through a bactericidal mechanism, rather than a bacteriostatic mechanism. 

### 2.3. Binding to Lipid Vesicles

Based on the proposed mechanism of membrane disruption, the ability of the C18G peptides to interact with lipid vesicles was investigated. All of the sequences contain a Trp residue which can be used as a reporter of the local environment. As the peptide moves from the aqueous milieu to the bilayer surface, the Trp experiences a significant change in the polarity of the local environment. This change in environmental polarity results in a shift of the Trp emission spectrum from more red-shifted (aqueous) to more blue-shifted (bilayer bound) [32]. Similarly, the depth of the Trp in the bilayer also affects the emission spectrum, with increasing depth in the bilayer resulting in more extensive blue shifting [33]. The shift in the emission spectrum can be quantified by changes in maximum emission intensity (𝜆_max_) or by a shift in the spectral barycenter [32,34,35].

**Figure 2:**
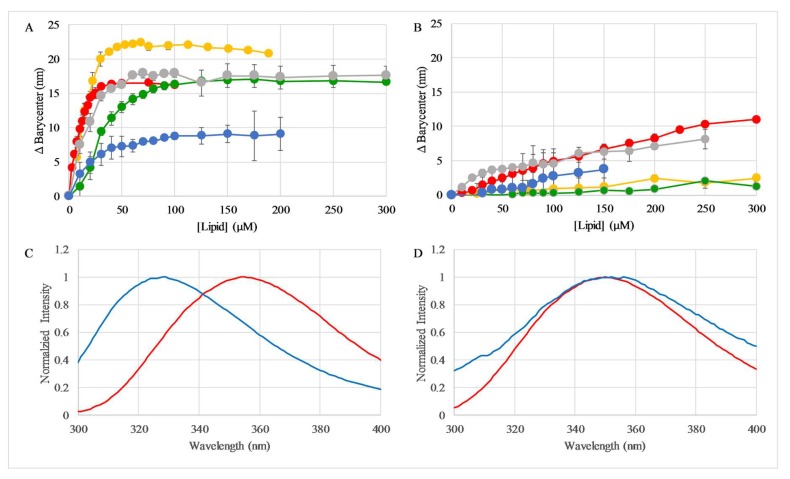
Trp fluorescence emission to measure peptide binding to vesicles. Samples containing 2 µM peptide in phosphate buffered saline (PBS) were titrated with either (**A**) 3:1 PC/PG or (**B**) 100% PC lipid vesicles. Trp emission barycenter was calculated after each addition of vesicles and plotted as a function of total lipid concentration. In both panels, colors represent C18G (green), C18G-Arg (red), C18G-His (gray), C18G-Orn (orange), C18G-Dap (blue). All data is the average of 2–5 replicates, and error bars represent standard deviations. Representative normalized spectra of C18G from panels (**A**,**B**) are shown in (**C**,**D**), respectively. In both (**C**) and (**D**), red spectra represent Trp emission in the absence of lipid, blue represents spectra when bound to 300 µM lipid. All data presented is after background subtraction and dilution correction when appropriate.

As judged by the shift in the emission barycenter, the peptides all exhibited the ability to bind to lipid vesicles that were composed of 3:1 1,2-dioleoyl-sn-glycero-3-phosphocholine/1,2-Dioleoyl-sn-glycero-3-phosphoglycerol (PC/PG) lipids (Figure 2A). The PG lipid headgroup contains a net negative charge, mimicking the net anionic surface charge of bacterial cells. In all cases, the peptides exhibited a significant change in the emission spectrum upon addition of lipid vesicles. There was considerably weaker binding to vesicles composed of 100% PC lipids, consistent with the electrostatic driving force in AMP selectivity, as well as previously published results [32,34]. Representative Trp emission spectra for the peptide in the unbound and fully-saturated lipid bound states are shown in Figure 2C,D. 

### 2.4. Secondary Structure Analysis by Circular Dichroism Spectroscopy

Many different AMPs have been shown to adopt an alpha-helical structure when bound to lipid bilayers or bilayer mimetics [36,37]. This helical structure is thought to play an important role in the mechanism of action of the peptides, allowing the formation of the facially amphiphilic structure in which the hydrophobic amino acids segregate to one face of the helix. This structure facilitates the insertion of these hydrophobic groups into the bilayer, and thereby, induce membrane disruption. 

**Figure 3:**
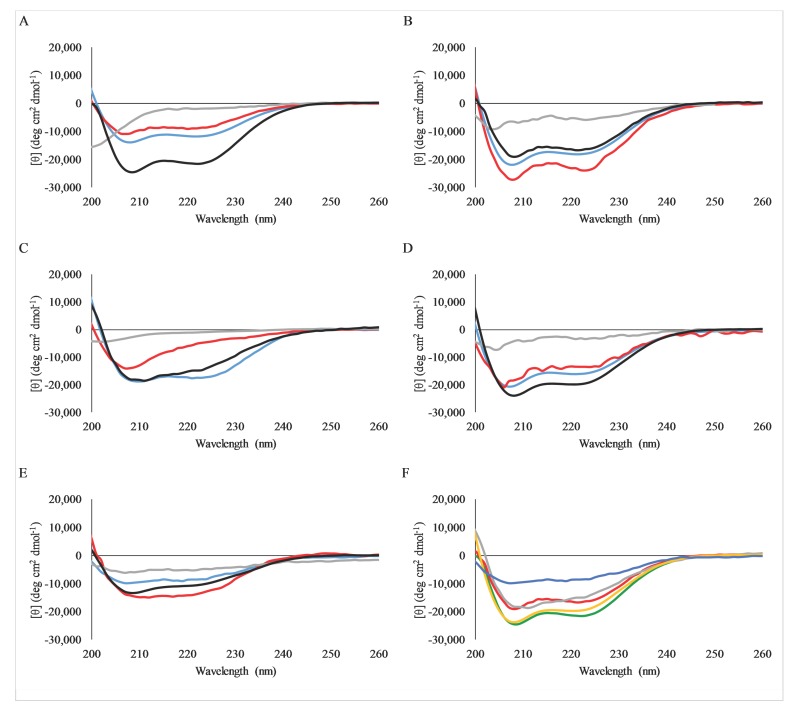
Circular dichroism spectra of peptides in varying environments. Spectra of samples containing 5 µM peptide in PBS (gray), 1:1 PBS/trifluoroethanol (red), 10 mM SDS in PBS (blue), or 3:1 PC/PG lipid vesicles (black) are shown in panels A–E: (**A**) C18G, (**B**) C18G-Arg, (**C**) C18G-His, (**D**) C18G-Orn, (**E**) C18G-Dap. (**F**) Comparison of all peptides when bound to lipid vesicles: C18G (green), C18G-Arg (red), C18G-His (gray), C18G-Orn (orange), C18G-Dap (blue). All data is the average of 64 scans, with background spectra subtracted.

Circular dichroism spectroscopy can be used to quickly determine the secondary structures that AMPs adopt. Due to their relatively short length, AMPs, including C18G, cannot form higher order tertiary structures. Thus, CD spectra of these peptides can usually give good insight into the structural conformations adopted under different conditions. The CD spectra of the C18G cationic variants are shown in Figure 3. In all cases, the peptides exhibited very weak spectra in buffer corresponding to a small fraction of ⍺-helical structure or random coil. In contrast, all peptides exhibited the canonical spectral signatures, indicating alpha-helix formation when mixed with 100 mM SDS (a membrane mimetic) or with 3:1 PC/PG lipid vesicles. This trend is identical to that reported for the parent C18G peptide [32]. Notably, the C18G-Dap peptide exhibited somewhat less helical character compared to the other peptides, which has been reported previously [38]. A comparison of the spectra of all peptides bound to PC/PG vesicles is shown in Figure 3F. 

### 2.5. E. coli Membrane Permeabilization

The ability of all the C18G cationic variants to bind lipid vesicles and exhibit antimicrobial activity against some, if not all, of the bacteria tested indicates that a membrane disruption mechanism of action may be involved. While there are numerous liposome leakage assays that can measure content leakage or pore formation, they have numerous drawbacks. Primarily, the liposome systems lack the diversity in lipid types, acyl chain heterogeneity, extracellular polysaccharides, and membrane proteins that are found in bacteria. Using an enzyme/chromophoric substrate system allows for direct measurement of leakage across the membrane of intact bacteria, retaining all of the normal surface charge and functional group heterogeneity found under normal conditions. Briefly, these assays rely on a bacterially expressed enzyme and a membrane-impermeant chromogenic substrate. Under normal conditions, transport of the substrate across the bilayer(s) would be slow, and thus, conversion to the colored product is slow. However, if the peptides (or other compounds) disrupt the bilayer integrity, then the substrate can pass across the bilayer(s) more readily, resulting in an increased rate of conversion, as measured by increases in absorbance.

**Figure 4:**
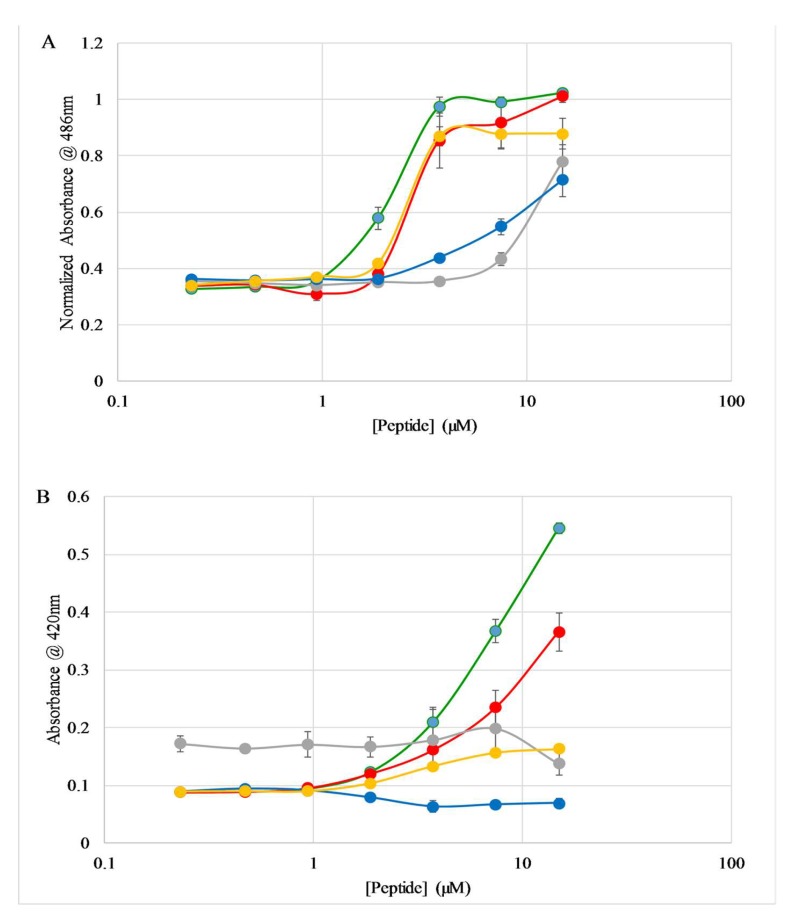
*E. coli* membrane permeabilization. Chromogenic substrate breakdown after 30 min of exposure to peptides. (**A**) Outer membrane permeabilization assayed by nitrocefin breakdown. (**B**) Inner membrane permeabilization assayed by ONPG breakdown. In both panels colors represent C18G (green), C18G-Arg (red), C18G-His (gray), C18G-Orn (orange), C18G-Dap (blue). All data is the average of at least 3 replicates, where error bars represent the standard deviation.

Permeabilization of the *E. coli* outer membrane is monitored using the periplasmic enzyme β-lactamase and the chromogenic substrate nitrocefin. A summary of the results of the outer membrane permeabilization experiments are shown in Figure 4A. The C18G, C18G-Arg, and C18G-Orn peptides exhibited a traditional dose-dependence profile, with near maximal permeabilization observed above 3.75 µM. The C18G-Dap peptide induced permeabilization to a lesser extent over this concentration range, while the C18G-His sequence induced almost no permeabilization of the outer membrane except at 15 µM, the highest concentration of peptide tested. The full time course of the permeabilization assay is shown in the supplementary files (Figure S1). 

Permeabilization of the *E. coli* inner membrane is monitored with the cytoplasmic enzyme β-galactosidase and the chromogenic substrate ortho-nitrophenyl-β-galactoside (ONPG). This assay in conceptually similar to the outer membrane assay described above, but is focused only on the permeability of the inner membrane. The results are shown in Figure 4B for the 30 min time point of the assay, and the full time course is shown in the supplementary figures (Figure S2). Similar to the results for the outer membrane, the C18G parent peptide exhibited the highest degree of permeabilization. However, the C18G-Arg induced less leakage in this case, and the C18G-Orn induced very little observed leakage. The C18G-His and C18G-Dap peptides induced little to no permeabilization as judged by this assay.

### 2.6. S. aureus Membrane Permeabilization

The membrane architecture varies greatly between Gram-positive and Gram-negative bacteria. The ability of the peptides to permeabilize a Gram-positive bacterial membrane was also investigated; however, in this case, the use of a bacterial enzyme and chromogenic substrate was not possible. Instead, membrane permeability was assessed using the DNA binding fluorophore propidium iodide (PI). The PI molecule is generally impermeable across the cell membrane under normal conditions, but upon permeabilization, PI can readily cross the membrane and bind to DNA, resulting in a dramatic increase in fluorescence emission intensity.

**Figure 5:**
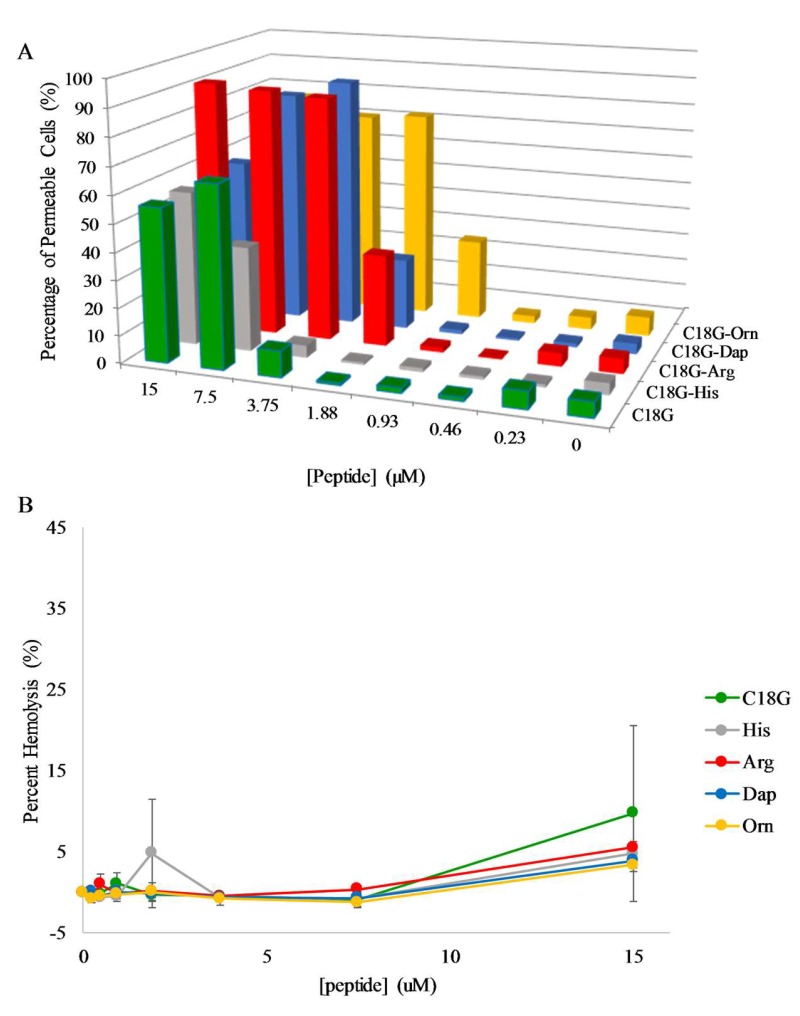
Membrane permeabilization. (**A**) Permeabilization of *S. aureus* membranes by peptides. Cultures of *S. aureus* were incubated with varying concentrations of peptide and 3.75 µg/mL propidium iodide (PI). PI fluorescence was measured via flow cytometry after 30 min of exposure to peptides. Percent leakage was determined based on the positive control CTAB (data not shown). (**B**) Peptide induced hemolysis. Defibrinated sheep red blood cells were incubated for 1 h with varying concentrations of peptide or control at 37 °C in sterile PBS. After pelleting the remaining cells, the absorbance of the supernatant was measured at 415 nm to detect released hemoglobin. Percent leakage was determined based on the positive control CTAB (Figure S3). All data is the average of at least 3 replicates, where error bars represent the standard deviation.

Flow cytometry was utilized to monitor the PI leakage across the *S. aureus* membrane and resultant fluorescence emission changes. The results of these experiments are shown in Figure 5A. Overall, the same general pattern of dose dependent permeabilization behavior was observed for *S. aureus* as were seen for the outer membrane of *E. coli*. However, the more detailed analysis shows some interesting differences between Gram-positive and Gram-negative permeabilization profiles. First, the C18G parent peptide was not the most active in permeabilizing the *S. aureus* membrane, instead the C18G-Arg peptide induced ~90% leakage down through 3.75 µM, while the C18G-Orn also induced >50% leakage through 1.88 µM. Second, the C18G-His induced >50% leakage at 15 µM and 7.5 µM, which is in contrast to the *E. coli* results and the MIC experiments, where this peptide was generally inactive.

### 2.7. Hemolysis

Considering the membrane-active mechanism of many AMPs and the work presented earlier, it became evident that the membrane permeabilizing action against mammalian cells was also important to investigate. Using a well characterized hemolysis assay, the ability of the peptides to disrupt or damage sheep red blood cells (RBCs) was measured. Briefly, the peptides are mixed with defibrinated blood, incubated, and then centrifuged to collect the intact RBCs. The absorbance of the supernatant is then measured at 415 nm, corresponding to the absorbance of hemoglobin. If the peptides disrupt the membranes, hemoglobin leaks out of the damaged RBCs into the supernatant. Percent hemolysis is calculated in comparison to an untreated control (0%) and a control treated with 8 mM CTAB (100%). The results are shown in Figure 5B. Overall, the peptides induced hemolysis to a very low extent, with the greatest hemolysis caused by the C18G sequence (~9%) at the highest concentration tested (15 µM). The hemolysis induced by the control compound is shown in Supplemental Figure 3.

## 3. Discussion 

AMPs are a widely studied system, due to the diversity of sequences found in nature, high specificity, and broad-spectrum activity, and relative ease of synthesis. However, despite several decades of work on these peptides, no clear consensus sequences are evident, nor are there tools that can accurately predict or de novo design AMPs. Investigating these molecules from a more traditional structure–activity relationship approach may help yield insights into the molecular mechanisms that are important for function, and/or provide information translatable to the design of novel small molecule therapeutics that retain the beneficial qualities of AMPs.

In most cases investigated here, the specific identity of the cationic side chains did not have a significant effect on the behavior of the peptide. In almost all cases, the cationic variant peptides retained similar antibacterial activity to the parent sequence, differing no more than 2-fold in MIC for any single bacterial species (Table 2). This is supportive of the hypothesis that the cationic charge of the peptide is mainly involved in the initial binding event between the peptide and the bacterial cell surface. However, there are clear nuances in the data that are important in the full understanding of the structure–activity relationship in these AMPs.

One area of interest is the non-naturally proteinogenic amino acids used in this study, Orn and Dap. Non-natural and non-proteinogenic amino acids have been of significant interest in both the peptide design and pharmaceutical fields [39]. From the peptide design perspective, these amino acids offer additional routes of studying structure–activity relationships, while from the medicinal chemistry perspective, they provide a method of introducing functional groups that may be less susceptible to natural proteolytic degradation or recognition by the immune system [37,40]. The hypothesis that peptide penetration into the hydrophobic core of the bilayer is critical for membrane disruption and peptide activity is therefore directly linked to side chain length, and thus, the maximum possible depth the peptide can penetrate into the bilayer. The results presented here indicate that the use of these amino acids as the cationic functionality does not significantly impact the antibacterial activity (Table 2). However, in the case of C18G-Dap, the ability of the peptide to adopt an alpha-helix was diminished, likely due to the close proximity of the charged side chain amine group to the backbone of the peptide, as reported previously [38,41]. These findings are especially interesting because the effect of shortening the cationic side chain is much less pronounced than that observed for the shortening of the hydrophobic side chains in peptides or peptidomimetic polymers [32,42]. However, these results contrast with the work of Palermo et al., who demonstrated that shortening the cationic side chains in antimicrobial polymers had significant effects on antimicrobial activity [43]. One possible explanation is the length difference in the polymer side chains in their study (C6–C2) present a slightly larger difference in length than studied in the peptide system (C4–C1). Alternatively, the fact that the peptide still adopts some helical conformation, judged by the CD spectra in Figure 3, may allow it to penetrate deeper into the nonpolar bilayer core than that of an unstructured polymer [43]. Nonetheless, the antimicrobial activity is more tolerant to changes in the length of the spacer in the cationic residues than the hydrophobic residues, and thus, can potentially be exploited in next generation peptide or peptidomimetic designs.

The identity of the functional group that imparts the cationic charge to the sidechain had somewhat more pronounced effects. The Lys, Orn, and Dap side chains all contain primary amine groups, while the Arg contains a guanidino group, and His contains an imidazole ring. The comparison of these side chains is complicated by the differences in size/length, as well as the differences in the pK_a_ of the side chains. The guanidino group had no significant effect on the activity of the peptide, even though similar functional groups improved activity in peptidomimetic arylamide foldamers [44,45]. The bidentate nature of the Arg guanidino group is thought to enhance the binding to the bacterial membrane [46,47]. A modest enhancement of binding was observed comparing C18G to C18G-Arg; however, it is unlikely that this is due to the amine vs guanidino difference, as the C18G-Arg had similar binding behavior to C18G-Orn (amine) and C18G-His (imidazole). Additionally, the inclusion of Arg in short peptides has been linked to the ability to cross bilayers without significant bilayer disruption, as seen in cell penetrating peptides (CPPs) [48,49]. Based on the data, inclusion of Arg alone does not impart the ability to cross bilayers without disruption, as seen from the C18G-Arg peptide disruption of both *E. coli* and *S. aureus* membranes (Figure 4 and Figure 5A). Notably, the C18G-Arg was the most effective at disrupting *S. aureus* membranes, indicating there may be differences in peptide–lipid interactions as a result of the different lipid compositions in the two bacteria. The data does show a significant increase in binding to zwitterionic PC membranes compared to C18G, and all of the peptides that contain primary amines as the cationic group. While the translocation of C18G-Arg was not directly measured in comparison to the other peptides, the molecular discriminators between CPP and AMP may also be linked to the non-cationic residues, which in the case of C18G, is primarily hydrophobic groups. 

Regarding His, at pH 7, the His side chains are neutral, thus removing the electrostatic driving force for binding the anionic membrane, which is consistent with the diminished antibacterial activity of the C18G-His peptide. Nonetheless, the peptide was able to bind to model lipid vesicles and induce some degree of membrane permeabilization. This binding is likely driven through the hydrophobic forces and/or the polar uncharged imidazole with the anionic lipid headgroups, as well as the remaining +1 charge on the peptide from the N-terminus. Interestingly, the C18G-His peptide exhibited a small, but reproducible decrease in permeabilization of *E. coli* membranes at the highest concentrations tested. This may indicate that the C18G-His is forming an oligomeric structure at higher concentrations, which competes with membrane binding. This type of His-driven oligomerization at neutral pH has been previously reported [50]. The work by Marquette et al. and by many other groups on His-containing AMPs clearly show a pH dependence in peptide behavior and activity that would be of great future interest in the development of the C18G peptide platform [51,52].

It should also be noted that the C18G parent sequence, and thus, all the derivative sequences studied herein, are somewhat unique in that they contain only Leu residues as the hydrophobic groups. The original development of the C18G sequence was based on the C-terminal fragment of platelet factor IV, and was modified to increase activity [27]. However, naturally occurring AMPs often have a mixture of hydrophobic amino acids in the sequence, and the membrane disruption by AMPs and mimetics has been clearly linked to the overall hydrophobicity of the molecule [13,42,53,54]. Additionally, a number of groups have demonstrated the position of both cationic and hydrophobic residues can have significant impacts on the activity and selectivity of AMPs [55,56,57,58,59]. Taken together, these findings may necessitate a much finer-grained approach to detailed structure–activity relationship studies in AMPs. The role of the cationic residues in the context of specific hydrophobic amino acids may also impact activity, such that the tolerance of cationic chain lengths may be significantly influenced by the size and shape of the corresponding hydrophobes in the molecule. 

Overall, the results show that there is flexibility in the cationic residues in antibacterial peptides. The binding of the peptides to lipid bilayers is clearly influenced by electrostatic interactions in that peptides bind to bilayers containing anionic lipids with higher affinity than zwitterionic bilayers. Even the C18G-His peptide which has lower net charge at pH 7 compared to the other sequences bound anionic bilayers with higher affinity than zwitterionic bilayers. However, the results also show that binding to anionic bilayers does not inherently translate to antibacterial activity. This is not surprising as there are numerous examples of peptide and protein sequences that bind to bilayers but do not disrupt bilayer integrity [58,60,61,62]. A better predictor of antibacterial efficacy is the membrane permeabilization assays. The trends observed in the outer membrane leakage assay correspond to the trends observed in MIC analysis for *E. coli*, indicating these two properties may be linked. 

## 4. Materials and Methods 

### 4.1. Materials

Peptides were purchased from Anaspec (Fremont, CA, USA), Genscript (Piscataway, NJ, USA) or synthesized in-house by solid phase FMOC-chemistry methods. In-house synthesized sequences were synthesized on a CEM-Liberty microwave-assisted synthesizer using a rink-amide resin with DMF as the main solvent and 20% piperidine in DMF as the deprotecting agent. Cleavage of peptides from the resin was achieved using a standard mixture of 92.5:2.5:2.5:2.5 trifluoroacetic acid (TFA)/H_2_O/triisopropylsilane (TIPS)/ethanedithiol. Peptide products were separated from the resin via filtration and precipitation in ice-cold diethyl ether. All peptides were purified by reversed-phase HPLC (RP-HPLC) with a linear gradient of water and acetonitrile each containing 0.1% TFA. Separations were performed on a Jupiter 300 C4 column (Phenomenex). Peptide identity in the HPLC fractions were confirmed by MALDI-TOF MS analysis performed by Bruker Daltonics (Billerica, MA, USA). Lipids, (16:0–18:1) 1-palmitoyl-2-oleoyl-sn-glycero-3-phosphatidylcholine (POPC, PC), and (16:0–18:1) 1-palmitoyl-2-oleoyl-sn-glycero-3-phosphatidylglycerol (POPG, PG), were purchased from Avanti Polar Lipids (Alabaster, AL, USA), and stored as stocks in chloroform at −20 °C. The buffers used were PBS (150 mM NaCl, 50 mM Na_2_HPO_4_/NaH_2_PO_4_; pH 7.0), 10× diluted PBS (for circular dichroism (CD) measurements), or Z-Buffer (0.1 M Na_2_HPO_4_/NaH_2_PO_4_, 10 mM KCl, 1 mM MgSO_4_, 0.05 M β-mercaptoethanol, pH 7.0).

### 4.2. Bacterial Culturing

Each bacterial strain was streaked onto LB–Miller agar (BD-Difco, Franklin Lakes, NJ, USA) plates from a frozen stock collection (*E. coli* D31 [63] *S. aureus* ATCC: 27660, *B. subtilis* ATCC: 6051, *P. aeruginosa* PAO1 [17], and *A. baumannii* ATCC: 19606). After incubation to allow colony growth, plates stored in a refrigerator at 4 °C. Overnight cultures were inoculated with a single colony of each bacterial strain into fresh LB or Mueller Hinton broth (MH, BD-Difco) in sterile glass test tubes. Inoculated samples were placed in a 37 °C shaking incubator (250 rpm) for ~18 h. After the overnight incubation, a 1:200 dilution of the culture was made in fresh LB or MH for use in subsequent experiments.

### 4.3. Minimal Inhibitory Concentration/Minimal Bactericidal Concentration Assays

Bacterial growth inhibition was investigated using the standard minimal inhibitory concentration (MIC) assay [64]. Cultures of bacteria were grown as described above, subsequently diluted to ~10^5^ cfu/mL in MH media. Subsequently, 90 μL of this diluted culture was added to wells of a sterile 96-well plate containing serially diluted aliquots of the peptides for a final volume in the well of 100 μL. The plate was covered and incubated at 37 °C for 18 h. After incubation the OD_600_ was measured with a Spectramax M5 multimode plate reader. Next, the minimal bactericidal concentration (MBC) was determined by taking 1 μL of culture from each well on the plate corresponding to peptide concentrations at and above the MIC and plating on a fresh LB-agar plate. The plate incubated overnight at 37 °C, and MBC was determined from the presence or absence of growth on each spot. 

### 4.4. Fluorescence Spectroscopy

Measurements were performed on a JY-Horiba fluoromax4 instrument with emission and excitation slits set to 2.5 nm. For lipid binding/titration assays, samples were excited at 280 nm with emission measured over the range of 300–400 nm. All samples contained 2 μM peptide and were titrated with a lipid vesicle stock ranging in concentration from 1–2 mM. The barycenter of the spectrum is an intensity-weighted average over the wavelengths measured, and Δbarycenter is the difference between the barycenter of the sample lacking lipid, and that of a given sample [32,65]. Spectra were corrected for background and dilution.

Vesicles were prepared by drying appropriate volumes of lipid stocks in a glass test tube under N_2_ flow. Further removal of solvent was accomplished by subjecting the lipid film to vacuum for 1 h. The lipid film was then rehydrated in PBS while vortexing. This suspension was sonicated in a high-power bath sonicator (Avanti Lipids, Alabaster, AL, USA) for 20 min to yield small unilamellar vesicles (SUVs). Sonicated SUVs typically range in size from 20–60 nm in diameter [66,67].

### 4.5. Circular Dichroism Spectroscopy

CD spectra were collected using a Jasco J-815 spectropolarimeter. Samples for CD spectroscopy contained 5 μM peptide in 0.1× PBS or 0.1× PBS and either 200 μM lipid vesicles, 100 mM SDS, or 50% (*v*/*v*) trifluoroethanol. All spectra shown are the average of 64–128 scans and were corrected for backgrounds by subtracting the spectra of the samples lacking peptide. 

Lipid vesicles for CD spectra were created using the ethanol dilution method [33]. Lipid films were created as described above, but after vacuum the films were first dissolved in 10 μL of ethanol and vortexed vigorously while adding the appropriate volume of PBS. 

### 4.6. E. coli Outer Membrane Permeability Assay

Permeabilization of the *E. coli* outer membrane was performed as described previously [32]. Briefly, a single colony of *E. coli* D31 was inoculated in LB Broth (Difco) containing 100 mg/mL ampicillin and allowed to grow at 37 °C with shaking overnight. The culture was diluted 1:200 in LB-Amp and incubated at 37 °C until the OD_600_ was ~0.2. The culture was subsequently centrifuged at 2500 rpm for 15 min. The pellet was resuspended in an equal volume of PBS and added to a 96-well plate containing serially diluted peptides or polymyxin B (positive control). Immediately before reading, nitrocefin was added to the wells (final concentration 0.25 mg/mL) and mixed by pipetting. The sample absorbance was measured at 486 nm for 90 min. All assays were performed at least in triplicate.

### 4.7. E. coli Inner Membrane Permeability Assay

Inner membrane permeabilization was performed as described previously [32]. The procedure follows that of the outer membrane permeabilization assay, described above, with several modifications. Bacteria were grown overnight in LB media lacking Amp, and dilutions were made in LB supplemented with IPTG. Assay plates were prepared the same, except that cells were not centrifuged before the assay, and were added to the plate directly in the culture medium. Positive control for the inner membrane assay was the cationic detergent CTAB. The substrate was ONPG dissolved in Z-buffer added to a final concentration 0.6 mg/mL in the wells. The sample absorbance was measured at 420 nm for 90 min. All assays were performed at least in triplicate

### 4.8. S. aureus Membrane Permeability Assay

Flow cytometry was used to identify cell permeability by incorporation of propidium iodide (PI) into *S. aureus* under various concentrations of CTAB detergent, C18G, C18G-Arg, C18G-His, C18G-Orn, C18G-Dap peptides using a BD FACSCelesta flow cytometer. Approximately 10,000 cells per sample in 100 µL of PBS, permeabilization reagent, and PI 5.6 μM solution, were incubated for 30 min or 45 min at 23 °C counted in duplicate, and fluorescent signal was evaluated Ex 488 nm laser, Em 575 filter. Percentage of cells found to be permeable for all samples was established by gating around cells on histogram illustrating PI signal with known complete permeabilization CTAB 102 µM.

### 4.9. Hemolysis Assay

Defibrinated sheep blood was diluted 2-fold in sterile PBS and pelleted in a benchtop clinical centrifuge for 10 min. The pellet was resuspended in PBS to the original volume, and this process was repeated 3× such that the supernatant was no longer red/pink in color. The pellet containing RBCs was resuspended in PBS to the original volume. Next, 135 μL of RBCs was added to the wells of a round bottom 96-well plate, each well containing serially diluted peptides or the positive control CTAB. The plate was incubated at 37 °C with shaking for 1 h. The plate was then centrifuged to pellet the remaining RBCs and 6 μL of the supernatant was transferred to a flat bottom 96-well plate containing 94 μL PBS in each well. Absorbance was measured at 420 nm. Percent hemolysis was calculated based on the absorbance in each well after subtraction of the absorbance in wells containing no peptide, and then normalized against the absorbance in the highest concentration CTAB wells (8 mM) taken as 100% lysis.

## Figures and Tables

**Table 1:** Peptide Sequences.

Name	Sequence	Net Charge ^a^	MW (Da)	MW (Da) Found
C18G	ALWKKLLKKLLKSAKKLG	+8	2065.7	2065.4
C18G-Arg	ALWRRLLRRLLRSARRLG	+8	2261.8	2261.5
C18G-His	ALWHHLLHHLLHSAHHLG	+1 ^b^	2128.5	2128.2
C18G-Orn	ALWOOLLOOLLOSAOOLG	+8	1967.7	1967.3
C18G-Dap	ALWXXLLXXLLXSAXXLG	+8	1771.7	1771.1

^a^ Net charge includes the 7 cationic residues plus the free N-terminus. The C-terminus of the peptide is amidated; ^b^ The pK_a_ of the His side chain is 6.04, therefore ~90% of His side chains are predicted to be neutral at pH 7, where experiments were performed.

**Table 2:** Minimum Inhibitory Concentration (MIC) and Minimal Bactericidal Concentration (MBC) (µM).

	*E. coli*		*P. aeruginosa*		*A. baumannii*		*S. aureus*		*B. subtilis*	
	MIC	MIC	MBC	MIC	MBC	MBC	MIC	MBC	MIC	MBC
C18G	1.875	7.5	7.5	15	1.875	n.d.	1.875	3.75	1.875	1.875
C18G-Arg	1.875	7.5	7.5	>15	3.75	7.5	3.75	3.75	1.875	1.875
C18G-His	3.75	>15	>15	>15	>15	>15	15	15	1.875	>15
C18G-Orn	1.875	3.75	3.75	3.75	1.875	1.875	1.875	1.875	3.75	7.5
C18G-Dap	3.75	7.5	7.5	7.5	3.75	3.75	7.5	7.5	1.875	1.875

n.d.—not determined

## Supplementary Materials

The Supplementary Materials are available online.
