# Supplementary material for: Role of Cationic Side Chains in the Antimicrobial Activity of C18G
Source: Molecules. 2018 Feb 4;23(2):329. doi: 10.3390/molecules23020329 (PMC6017431; doi:10.3390/molecules23020329)
Supplement: Supplementary file 1 [file molecules-23-00329-s001.zip › Role of cationic side chains in the antimicrobial activity of C18G - SUPPLEMENTARY INFORMATION.docx]

Role of Cationic Side Chains in the Antimicrobial Activity Of C18G

Eric M. Kohn^† 1^, David J. Shirley†^1^, Luba Arotsky^1^, Angela M. Picciano^1^, Zachary Ridgway^1^, Michael W. Urban^1^, Benjamin R Carone^2^, and Gregory A. Caputo^1,2,*^

^1^ Department of Chemistry and Biochemistry, Rowan University, Glassboro NJ, USA; [kohne2@students.rowan.edu](mailto:kohne2@students.rowan.edu) (E.M.K.); [dshirley9@students.rowan.edu](mailto:dshirley9@students.rowan.edu) (D.J.S); larotsky@upenn.edu (L.A.); ap775@cornell.edu (A.M.P.); Zachary.ridgway@stonybrook.edu (Z.R.); urbanm@rowan.edu (M.W.U), caputo@rowan.edu (G.A.C)

^2^ Department of Molecular and Cellular Biosciences, Rowan University, Glassboro NJ, USA; [carone@rowan.edu](mailto:carone@rowan.edu) (B.R.C. - *orcid.org/0000-0002-9822-4218)*

^†^ These authors contributed equally to this work.

***** Correspondence: caputo@rowan.edu; Tel.: 856-256-4505; *orcid.org/0000-0002-4510-2815*


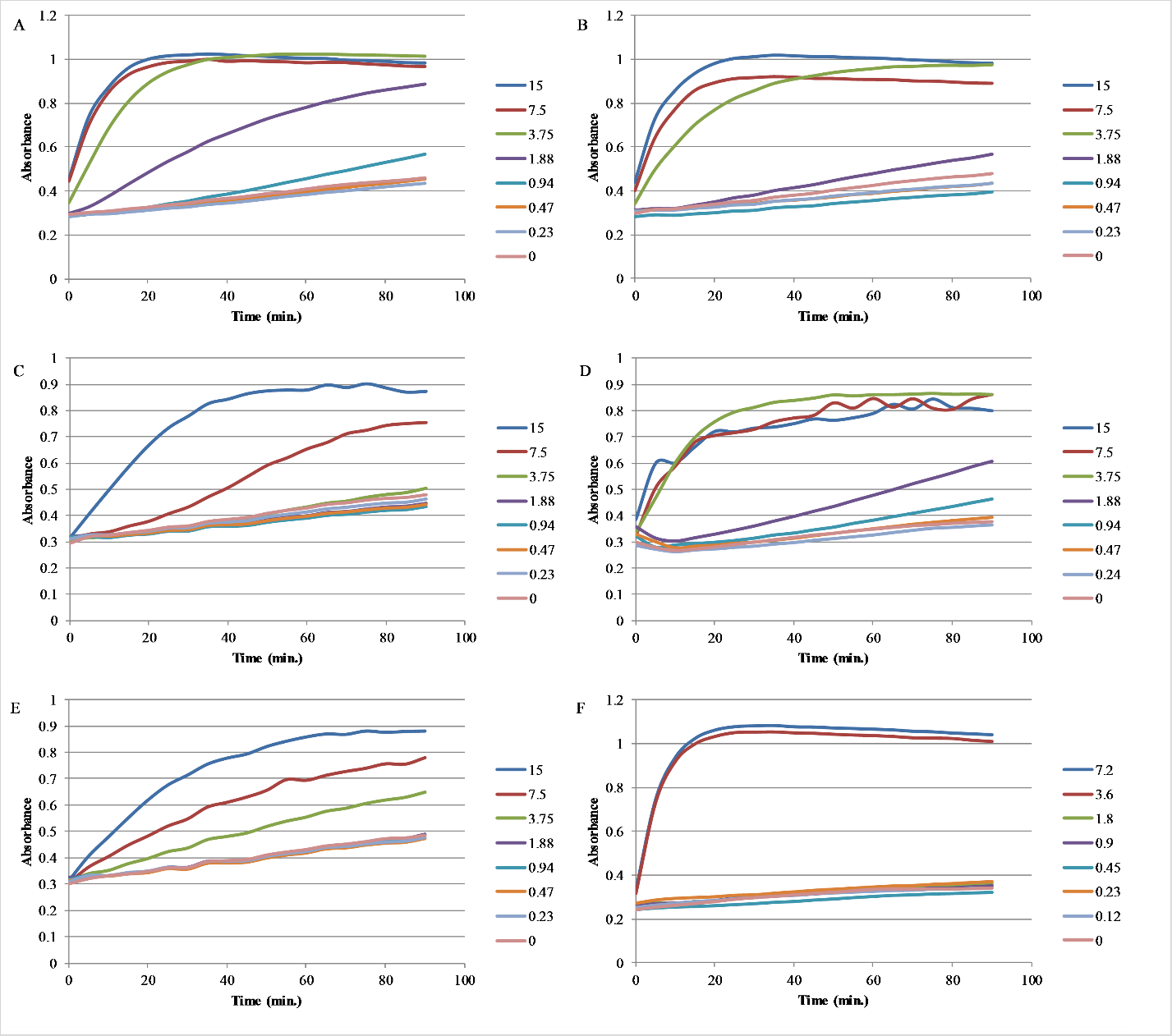


Supplemental Figure S1 – Time course of outer membrane permeabilization. Conversion of nitrocefin was monitored by absorbance at 486 nm. Measurements were taken in 5 minute intervals. Permeabilization was monitored after exposure to varying concentrations of (A) C18G, (B) C18G-Arg, (C) C18G-His, (D) C18G-Orn, (E) C18G-Dap, or (F) Polymyxin-B sulfate. Data shown are averages of 3 trials. All concentrations are shown in units of µM.


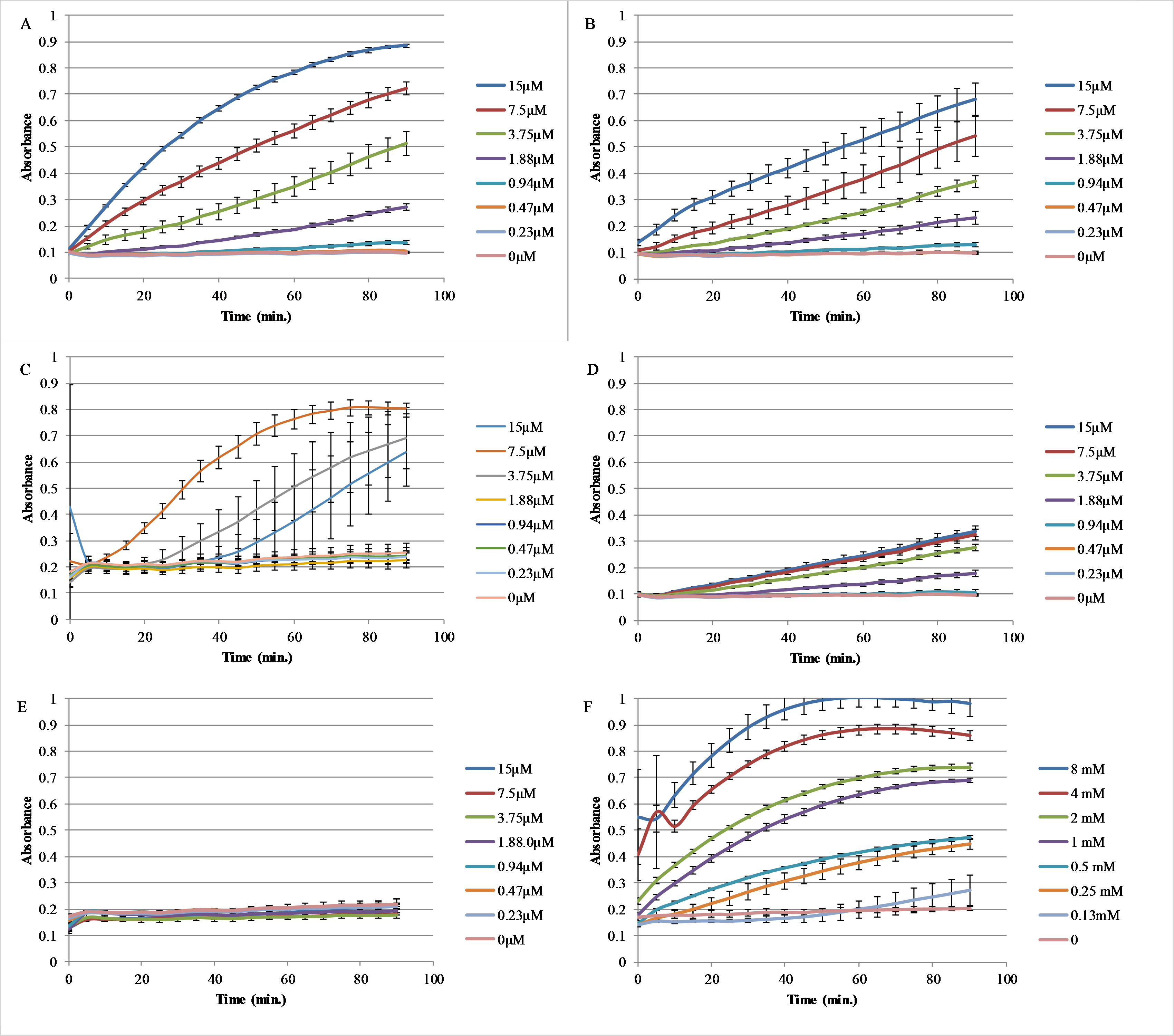


Supplemental Figure S2 – Time course of inner membrane permeabilization. Conversion of ONPG was monitored by absorbance at 420 nm. Measurements were taken in 5 minute intervals. Permeabilization was monitored after exposure to varying concentrations of (A) C18G, (B) C18G-Arg, (C) C18G-His, (D) C18G-Orn, (E) C18G-Dap, or (F) CTAB. Data shown are averages of 3 trials.


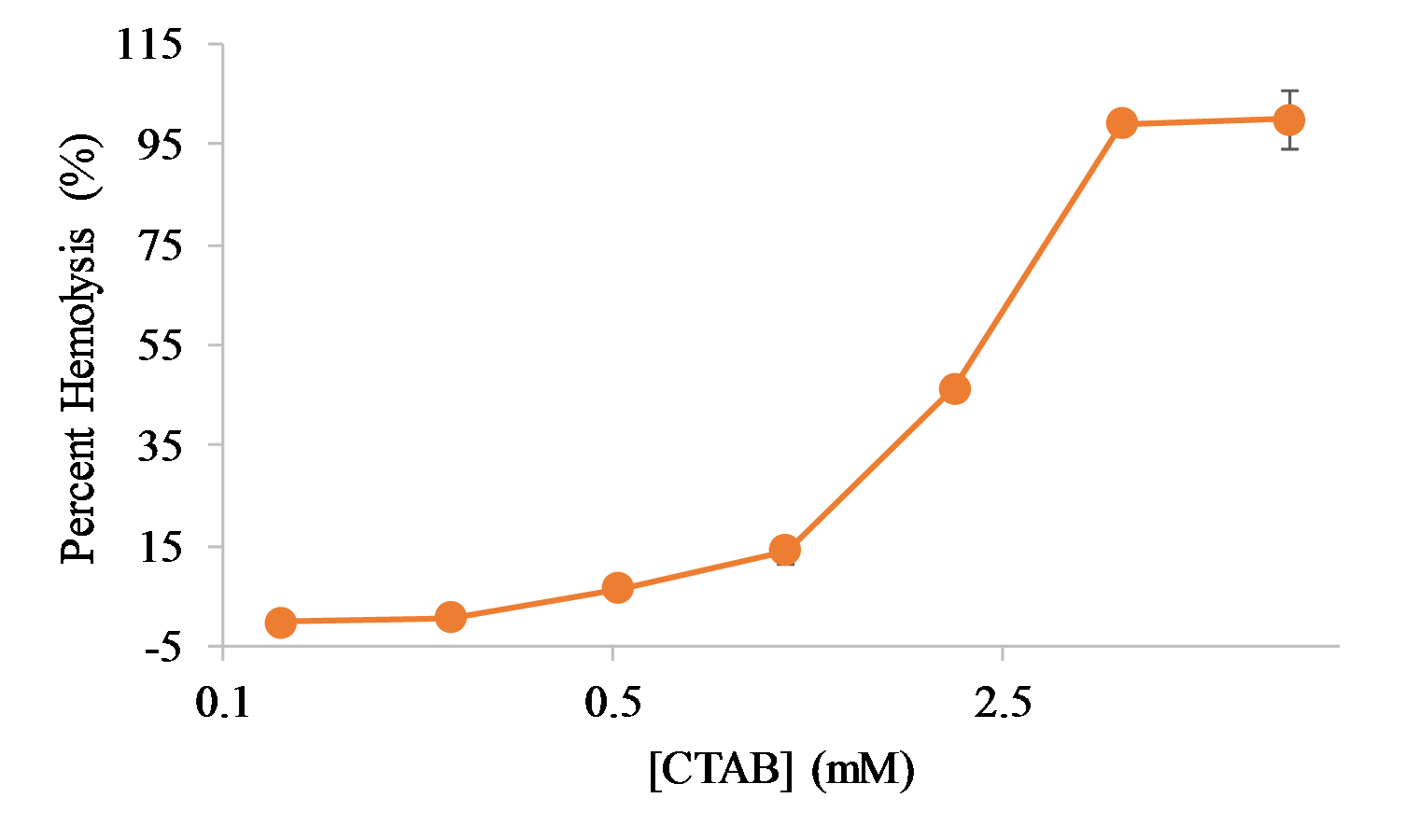


Supplemental Figure S3 – Control hemolysis assay using the cationic detergent CTAB. Defibrinated sheep red blood cells were incubated for 1 h with varying concentrations of peptide or control at 37 °C in sterile PBS. After pelleting the remaining cells, the absorbance of the supernatant was measured at 415 nm to detect released hemoglobin. All data is the average of at least 3 replicates where error bars represent the standard deviation.
