# Supplementary figures and images for: Role of Cationic Side Chains in the Antimicrobial Activity of C18G
Source: Molecules. 2018 Feb 4;23(2):329. doi: 10.3390/molecules23020329 (PMC6017431; doi:10.3390/molecules23020329)

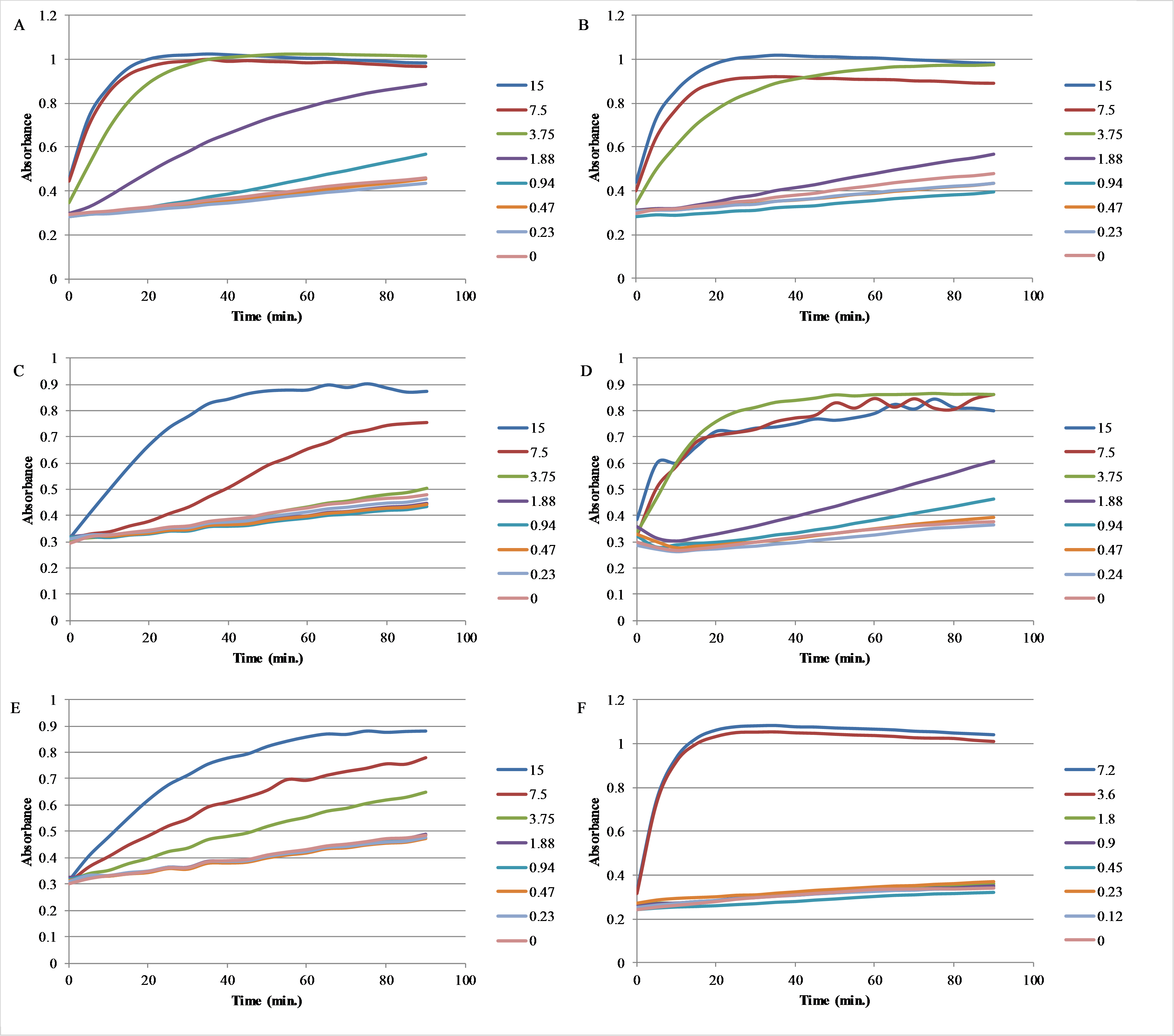

Supplement: Supplementary file 1 [file molecules-23-00329-s001.zip › CationicsS1.png]

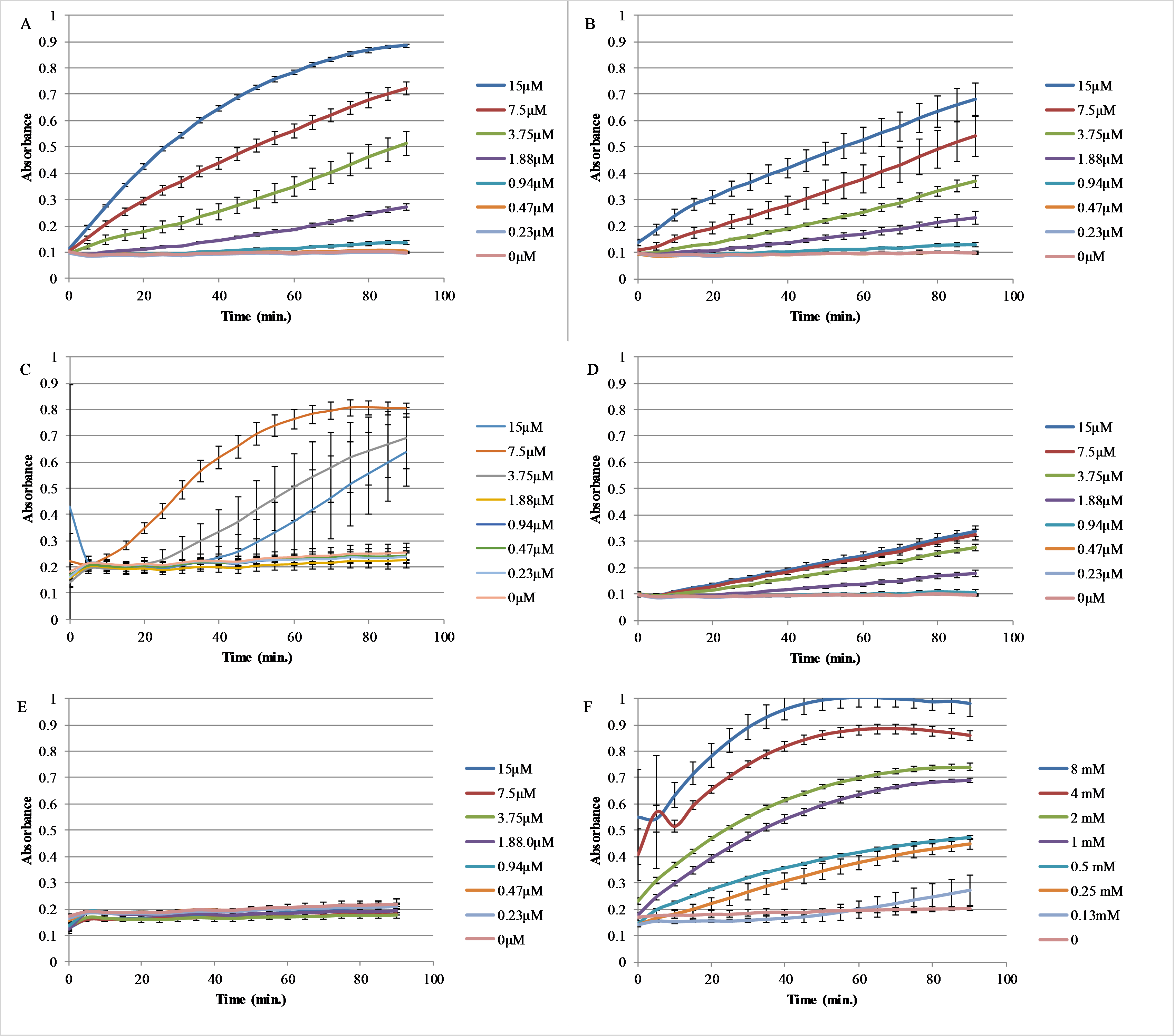

Supplement: Supplementary file 1 [file molecules-23-00329-s001.zip › CationicsS2.png]

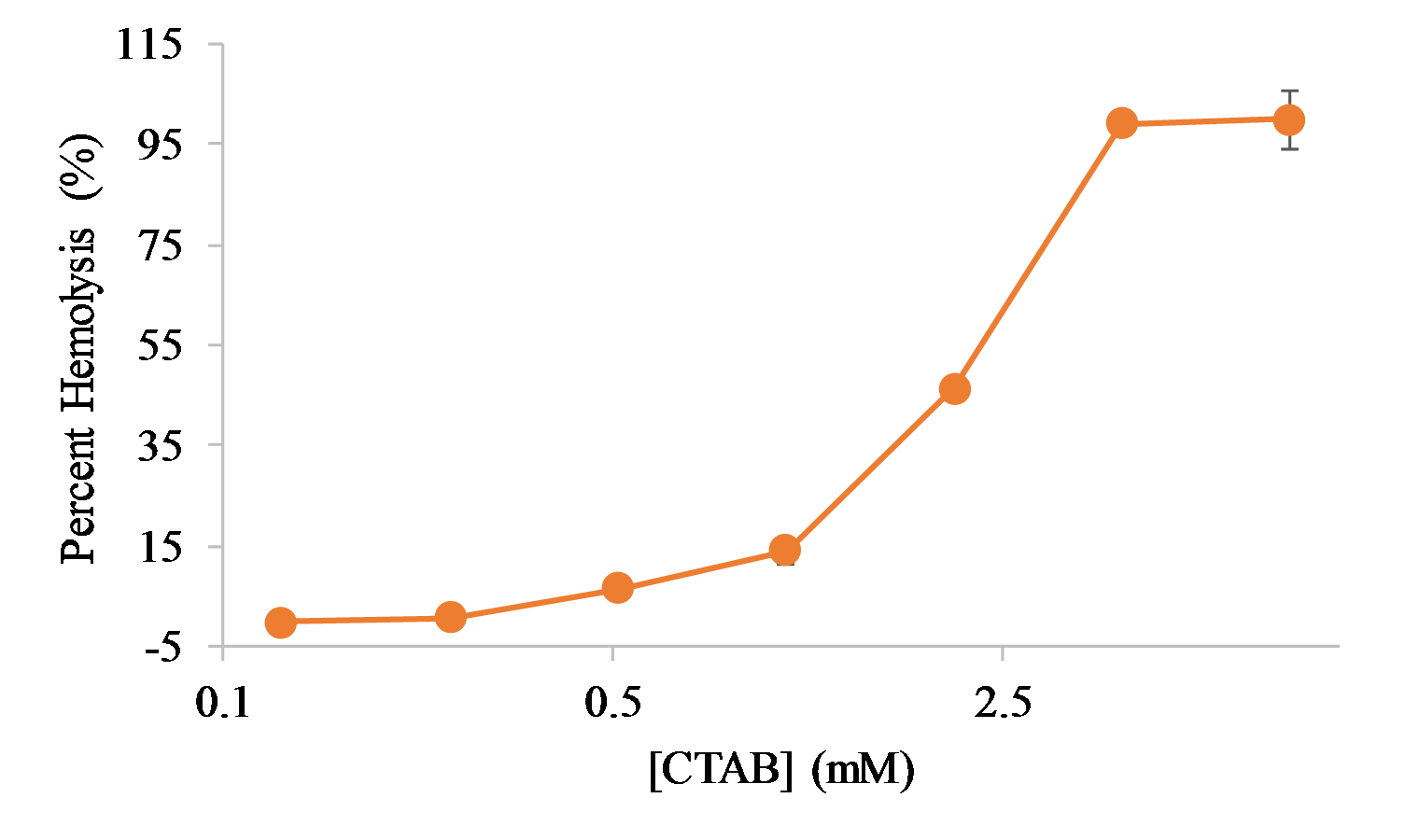

Supplement: Supplementary file 1 [file molecules-23-00329-s001.zip › CationicsS3.png]
